# Geographic distribution of *Orientia tsutsugamushi* strains on chigger mites in the Republic of Korea (2021–2023)

**DOI:** 10.1186/s13071-025-06839-3

**Published:** 2025-05-27

**Authors:** Hyeon Seung Lee, Byung-Eon Noh, Hyunwoo Kim, Heeil Lee

**Affiliations:** Division of Vectors and Parasitic Diseases, Korea Disease Control and Prevention Agency, 187, Osongsaengmyeong 2-Ro, Osong-Eup, Heungdeok-Gu, Cheongju, Chungbuk 28159 Republic of Korea

**Keywords:** Chigger mite, *Orientia tsutsugamushi*, 56-kDa type-specific antigen, Strains, Geographical distribution

## Abstract

**Background:**

Scrub typhus is caused by the larvae of chigger mites infected with *Orientia tsutsugamushi*, and many cases are reported globally. The virulence and prevalence of *O. tsutsugamushi* varies depending on the strain and region. Understanding the geographic distribution of *O. tsutsugamushi* strains is necessary for the prevention, control, surveillance, and future research on scrub typhus.

**Methods:**

Chigger mites were collected from wild rodents at 16 sites across the Republic of Korea (ROK) between 2021 and 2023. Molecular diagnosis of *O. tsutsugamushi* was performed on half of the collected chigger mites. After confirmation, sequencing and phylogenetic analysis were performed. To confirm the geographic distribution of *O. tsutsugamushi* strains in chigger mites, the ROK was divided into three regions on the basis of latitude and analyzed.

**Results:**

Overall, 135,204 chigger mites were collected from 1589 wild rodents. Half of the chigger mites were divided into 2928 pools for diagnosis of *O. tsutsugamushi* infection, of which, 152 pools were positive, resulting in a minimum infection rate of 0.22%. Phylogenetic analysis revealed six types of *O. tsutsugamushi* strains, including Karp-related (35.5%), Kato-related (17.8%), Boryong (15.8%), Saitama-related (15.1%), Gilliam-related (6.6%), and Simokoshi (1.3%). Additionally, strains exhibit distinct geographical distribution. The Karp-related strains were predominant and mainly distributed in the central region. Gilliam-related and Boryong strains were found in the northern, central, and southern regions, respectively.

**Conclusions:**

Our results demonstrate that the predominant *O. tsutsugamushi* strains in the ROK are Karp-related, with each strain being geographically separate. Changes in the geographic distribution, transmission routes, and other aspects of mite-borne diseases due to globalization and climate change will require continued surveillance and further research for prevention and control.

**Graphical Abstract:**

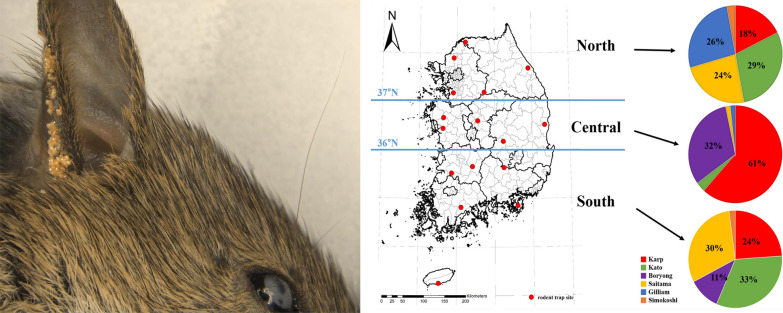

**Supplementary Information:**

The online version contains supplementary material available at 10.1186/s13071-025-06839-3.

## Background

Scrub typhus is one of the oldest vector-borne and zoonotic diseases globally, with chigger mite larvae infected by *Orientia tsutsugamushi* acting as a vector [1, 2]. After a short incubation period, *O. tsutsugamushi* causes various symptoms, such as headache, rash, fever, and chills [3–6]. The disease mainly occurs in the Asia–Pacific region, including the Republic of Korea (ROK), Japan, and China [1–5]. However, through globalization, cases were reported in other regions, including South America, Europe, and the Middle East [7–12]. Since scrub typhus was first reported in the ROK by the United Nations troops in 1951, it has been the most common mite-borne infectious disease, designated and managed as a legal infectious disease by the Infectious Disease Prevention Act Agency of the ROK since 1994 [13–16].

In the ROK, 14 genera and 60 species of chigger mites have been recorded, among which *Leptotrombidium pallidum*, *L. palpale*, *L. scutellare*, *L. orientale*, *L. zetum*, *Neotrombicula japonica*, *Euschoengastia koreaensis*, and *Helenicula miyagawai* are known to be vectors of *O. tsutsugamushi* [17]. Particularly, *L. pallidum* and *L. scutellare* are dominant vector species exhibiting unique geographical distribution patterns. *L. pallidum* is distributed throughout the ROK, whereas *L. scutellare* is mainly distributed in the southern regions such as Gyeongsang-do, Jeolla-do, and Jeju-do [18–20]. However, the distribution of *L. scutellare* has shifted further north owing to the effects of global warming [17, 21, 22].

The 56-kDa type-specific antigen (TSA) is an immunodominant protein located on the membrane surface of *Orientia* bacteria and shows considerable diversity due to mutations [23, 24]. On the basis of the antigenic properties of these 56-kDa TSAs, > 20 *O. tsutsugamushi* strains have been identified globally [25]. In addition to the three prototypes, i.e., Gilliam, Karp, and Kato, other strains were reported, including Shimokoshi, Kawasaki, and Kuroki [26]. In the ROK, native strains, such as Boryong, Jecheon, Yongworl, Wonju, and Pajoo, are also recognized [27].

The virulence of *O. tsutsugamushi* differs depending on the serotype and strain, and the prevalence of serotypes differs depending on the country or region [28, 29]. Several studies on differences in serum toxicity based on country or endemic area were conducted using mice [30–32]. *O. tsutsugamushi* strains vary depending on the country and endemic regions. In Asian countries, the Boryong strain is known to predominate in the ROK, the Gilliam strain in China, and the Karp strain in Thailand and Japan, while both the Karp and Gilliam strains predominate in Taiwan [28, 33–37]. In the ROK, studies have been limited to specific endemic areas rather than national surveys [27, 29, 38–40].

Understanding the geographic distribution of scrub typhus through accurate monitoring and research is crucial to prevent and control the disease [28, 33]. This study conducted a survey to understand the infectious status of *O. tsutsugamushi* in chigger mites collected from wild rodents throughout the ROK.

## Methods

### Wild rodent and chigger mite collection

**Fig. 1:**
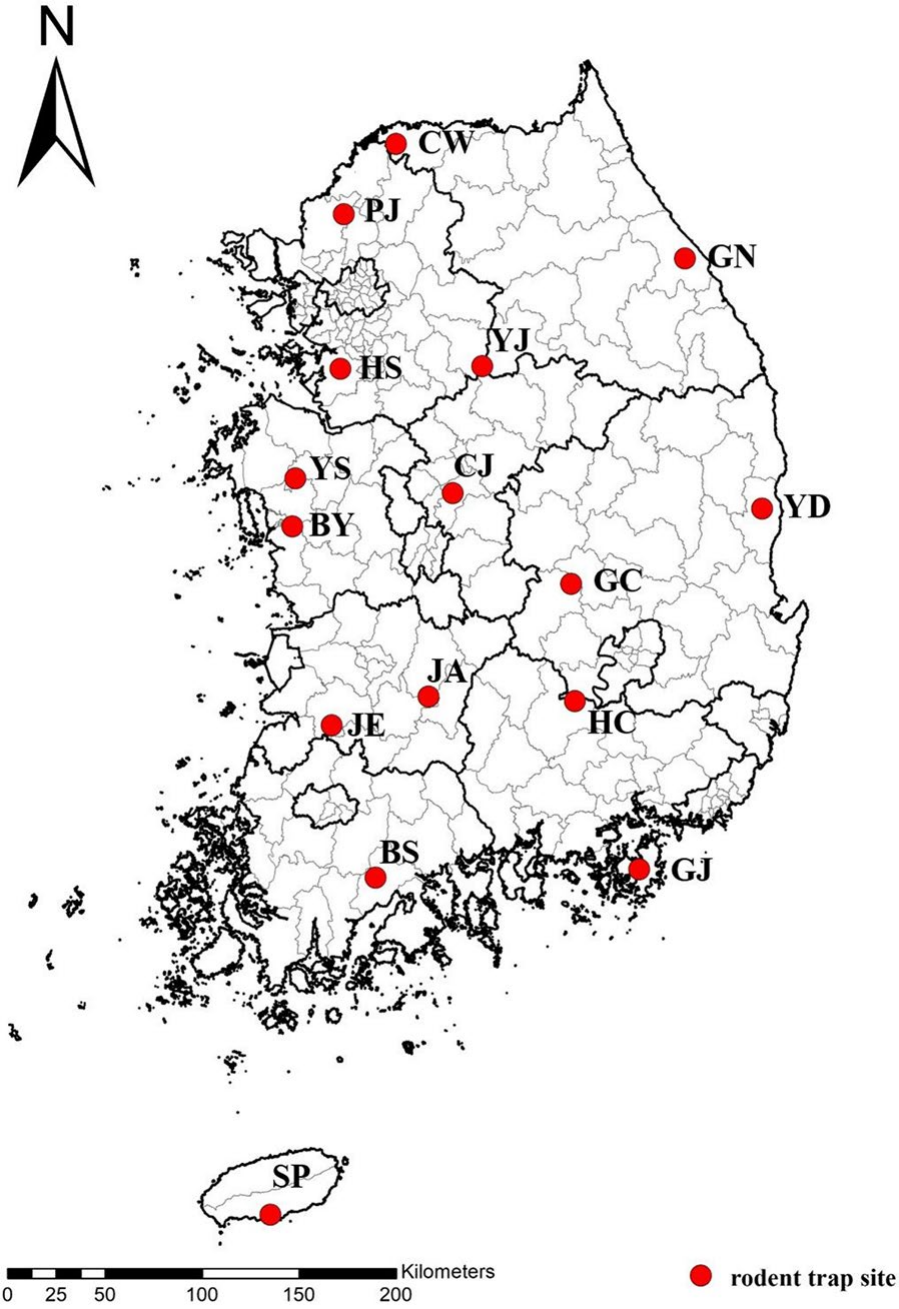
Wild rodent collection sites for chigger mites in the ROK in 2021–2023. *CW* Cheorwon-gun, Gangwon-do, *GN* Gangneung-si, Gangwon-do, *YJ* Yeoju-si, Gyeonggi-do, *GJ* Geoje-si, Gyeongsangnam-do, *HC* Hapcheon-gun, Gyeongsangnam-do, *GC* Gimcheon-si, Gyeongsangbuk-do, *YD* Yeongdeok-gun, Gyeongsangbuk-do, *HS* Hwaseong-si, Gyeonggi-do, *PJ* Paju-si, Gyeonggi-do, *JE* Jeongeup-si, Jeollabuk-do, *BS* Boseong-gun, Jeollanam-do, *JA* Jinan-gun, Jeollabuk-do, *SP* Seogwipo-si, Jeju-do, *CJ* Cheongju-si, Chungcheongbuk-do, *BY* Boryeong-si, Chungcheongnam-do, *YS* Yesan-gun, Chungcheongnam-do

Wild rodents were collected from 2021 to 2023 in spring (March and April) and autumn (October and November) from 16 areas in the ROK (Fig. 1). A total of five environments (mountains, reservoirs, fields, rice fields, and waterways) were designated in each area, and 100 Sherman live folding traps (H. B. Sherman Traps, Tallahassee, FL, USA) were used for collection, with 20 traps per environment at 10 m intervals. The traps were collected the next morning, and the captured wild rodents were euthanized using carbon dioxide and identified using a classification key [17]. The animal handling protocol was reviewed and approved by the Institutional Animal Care and Use Committee of the Korea Disease Control and Prevention Agency (KCDC-003–21, KCDC-154–23). Asphyxiated wild rodents were suspended upside down in a container with distilled water for 48 h. Using a dissecting microscope, only chigger mites were sorted and harvested. The collected chigger mites were counted, transferred to a 2-mL vial containing 70% ethanol, and stored at 4 °C for subsequent experiments.

### Molecular detection of *O. tsutsugamushi*

The molecular diagnosis of *O. tsutsugamushi* was performed on half of the collected chigger mites. In large-scale analyses of chigger mites, it is difficult to simultaneously identify individual chigger mite species and their infection rates because they are too small for identification. Hence, we proceeded by pooling 1–30 chigger mites per rodent for infection rate determination. After homogenization of the chigger mite pools using homogenization tubes (Bertin Technology, Montigny-le-Bretonneux, France), DNA was extracted using the G-spin Total DNA Extraction Kit (iNtRON Biotechnology, Seoul, Korea). For the molecular diagnosis of *O. tsutsugamushi*, nested PCR was performed using the LiliF™ TSUTSU Nested PCR kit (iNtRON Biotechnology) following the manufacturer’s protocol. Primers were designed specifically for the 56 kDa protein antigen gene of *O. tsutsugamushi*. The primer information is as follows: the first primer set: forward primer (5′-GCAATATTGCTAGTGCAATGTCTGC-3′) and reverse primer (5′-ATGCATGCATGRCGCTKCAATTTA-3′) and the second primer set: forward primer (5′-ATAGGCCTATAAGTATWGCKGATCG-3′) and reverse primer (5′-CATCTAGAYGCACTATTAGGCAAA-3′). The first PCR amplification was performed using a Thermal Cycler Dice (TaKaRa, Tokyo, Japan) with the first primer set under the following conditions: 94 °C for 5 min, 40 cycles of 94 °C for 30 s, 58 °C for 30 s, and 72 °C for 40 s, and a final extension at 72 °C for 5 min. The first PCR product (2 µL) was used as template for the second PCR, which was performed for 30 cycles under the same conditions as the first PCR. PCR products were visualized after separation by electrophoresis on a 1.5% (wt/vol) agarose gel. We calculated minimum infection rates (MIR; number *O. tsutsugamushi* positive chigger mite pools/number of tested chigger mites × 100).

### Sequence and phylogenetic analysis

The amplified PCR products were bidirectionally sequenced by Macrogen (Daejeon, Korea). The acquired sequences were edited using BioEdit v.7.2.6.1 and identified as the 56 kDa TSA gene via BLAST (http:///ncbi.nlm.nih.gov/blastn) search at the National Center for Biotechnology Information. The sequence information was registered in GenBank (accession number: PQ195687-826; *n* = 140). Phylogenetic analyses were analyzed using the maximum likelihood method with the General Time Reversible model in MEGA software version 10.0. This was repeated 1000 times for bootstrapping, and *O. chuto* strain Dubai (access number: LANP01000008) was used as the outgroup.

### Geographic distribution of *O. tsutsugamushi* strains

To determine the geographical distribution of *O. tsutsugamushi* strains in chigger mites, the ROK was divided into three regions on the basis of latitude. Latitudes above 37˚, 36–37˚, and below 36˚ were called the North, Central, and South, respectively.

## Results

### Wild rodent and chigger mite collection

Overall, 1589 wild rodents were collected over 3 years. The wild rodents collected included eight genera and 11 species. Among them, *Apodemus agrarius* was the most dominant species (1318; 82.9%), followed by *Crocidura spp*. (178; 11.2%) and *Micromys minutus* (36; 2.3%). In total, 135,204 chigger mites were collected from wild rodents. The overall chigger index (no. of chigger mites/rodent) was 85.1 (Table 1).

**Table 1:** Number of collected wild rodents and chigger mites from wild rodents in the Republic of Korea in 2021–2023

Species	No. of rodents (%)	No. of chigger mites	Chigger index^*^
*Apodemus agrarius*	1318	126,625	96.1
*Crocidura spp.*	178	420	2.4
*Micromys minutus*	36	1177	32.7
*Craseomys regulus*	23	2474	107.6
*Microtus fortis*	11	2655	241.4
*Apodemus peninsulae*	7	283	40.4
*Myodes regulus*	6	602	100.3
*Craseomys rufocanus*	5	882	176.4
*Tscherskia triton*	2	70	35
*Rattus norvegicus*	2	16	8
*Crocidura lasiura dobson*	1	0	0
Total	1589	135,204	85.1

^*^Chigger index—no. of chigger mites/no. of rodent

### Prevalence of *O. tsutsugamushi* in chigger mites

*Orientia tsutsugamushi* infections in chigger mites collected from 16 collection areas from 2021–2023 are summarized in Table 2. Detailed information is provided in Additional file 1: Table S1. Of the 135,204 chigger mites collected from 1589 wild rodents, 68,231 were divided into 2928 pools for molecular diagnostic experiments against *O. tsutsugamushi*. Of the 2928 pools, 152 were positive, with an MIR of 0.22% (152 pools/68,231 mites). Although a lower chigger index was confirmed in 2023, the highest number of positive pools was observed at 80, with the highest MIR at 0.34%.

**Table 2:** Summary of *Orientia tsutsugamushi* infection information for chigger mites collected from wild rodents in the Republic of Korea in 2021–2023

Year	No. of rodents infested by chigger mites/no. of rodents (%)	No. of chigger mites	Chigger index	No. of tested chigger mites (No. of pools)	No. of Ot^*^-positive chigger mite pools	MIR^**^
2021	375/471 (79.6)	40,215	85.4	20,024 (870)	33	0.16
2022	435/561 (77.5)	49,502	88.2	24,889 (1,040)	39	0.16
2023	434/557 (77.9)	45,487	81.7	23,318 (1,018)	80	0.34
Total	1244/1589 (78.3)	135,204	85.1	68,231 (2928)	152	0.22

^*^Ot *Orientia tsutsugamushi* *MIR* minimum infection rate of chigger mites (no. of *Ot*-positive chigger mite pools/no. of tested chigger mites × 100)

### Molecular and phylogenetic analyses

**Fig. 2:**
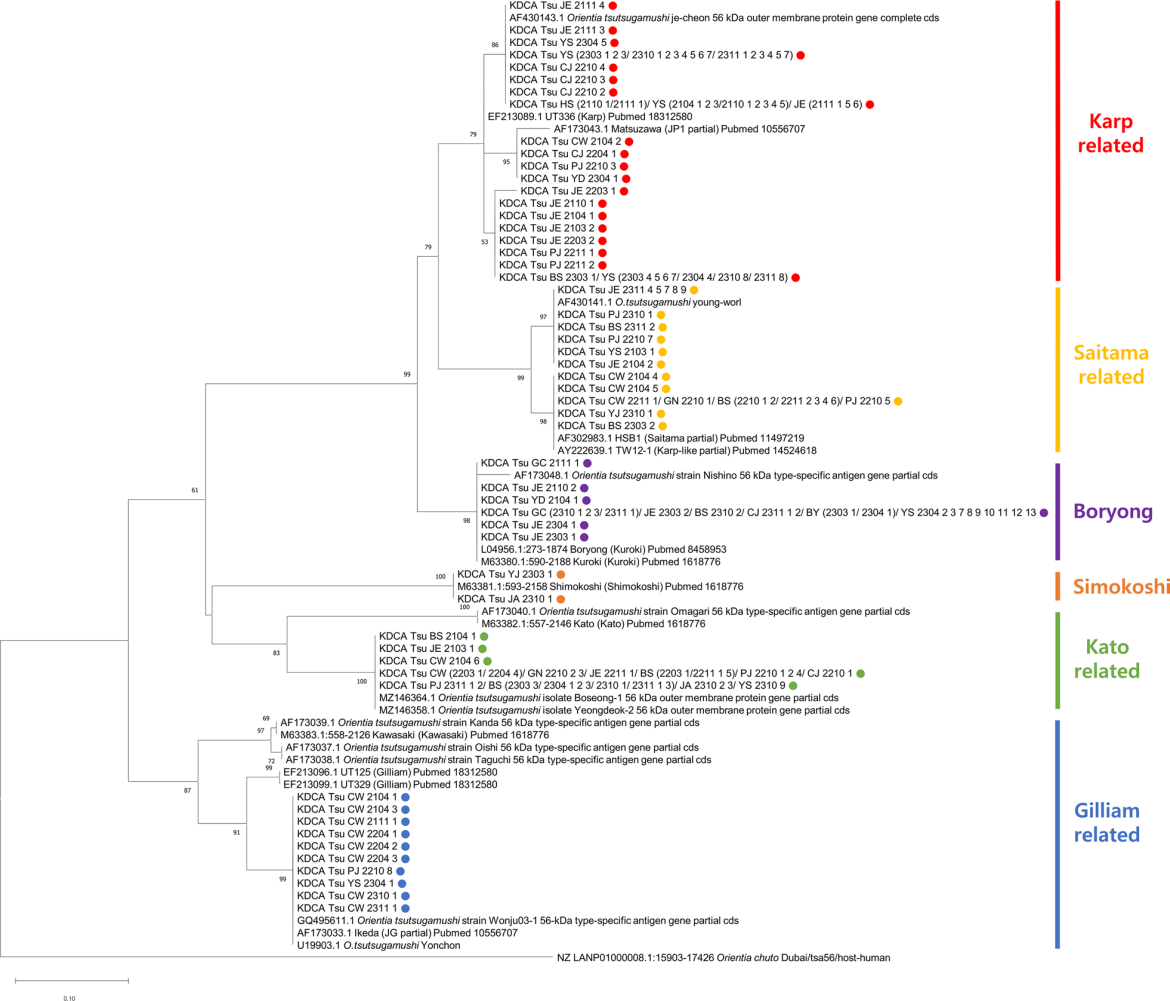
Annual phylogenetic tree analysis of *Orientia tsutsugamushi* from chigger mites collected from wild rodents at 16 collection sites in the ROK (2021–2023) based on the 56-kDa type-specific gene sequences. The maximum likelihood method was used in combination with bootstrap percentages for 1000 replicates, and *Orientia chuto* strain Dubai (access number: LANP01000008) was used as the outgroup. Identical sequences are abbreviated, and sequences detected in this study are shown as closed circles

**Fig. 3:**
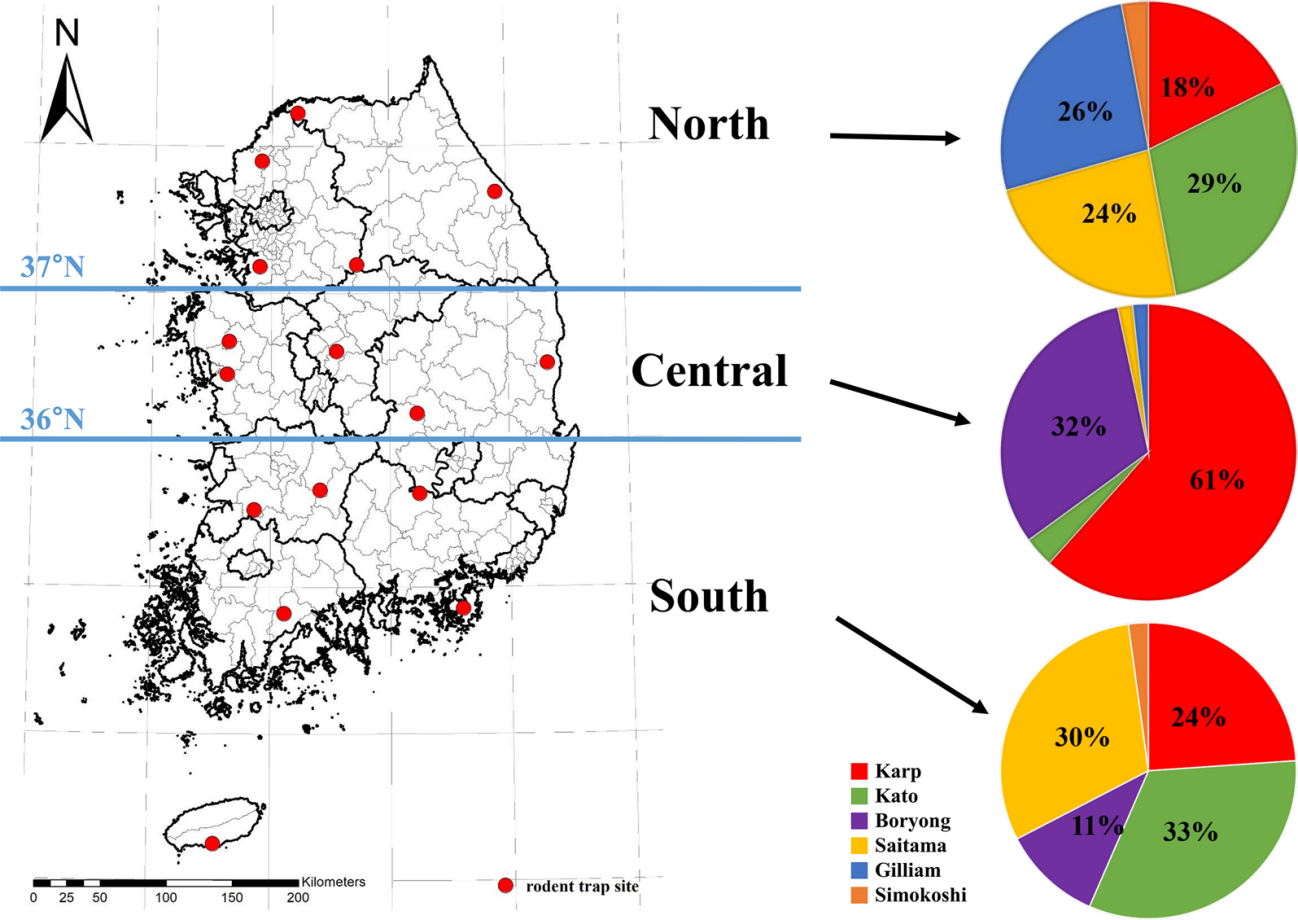
Geographic distribution of *Orientia tsutsugamushi* strains in chigger mites in the ROK in 2021–2023 (pie graph map)

Phylogenetic tree analysis was performed on the positive pools of *O. tsutsugamushi*. Among the 152 positive pools, 140 with clearly identified sequences were divided into six groups, along with previously registered reference sequences (Fig. 2). Karp-related strains (35.5%, 54/152) were the most common, followed by Kato-related (17.8%, 27/152), Boryong (15.8%, 24/152), Saitama-related (15.1%, 23/152), Gilliam-related (6.6%, 10/152), and Simokoshi (1.3%, 2/152). All six strains of *O. tsutsugamushi* were confirmed in *A.* *agrarius*, whereas Karp- and Giliam-related strains were confirmed in *Microtus fortis* collected from Cheorwon-gun. In *Cracseomys regulus* and *M. minutus* collected from Yesan-gun, Boryong and Karp-related strains were identified (data not shown). The geographical distribution of *O. tsutsugamushi* strains was confirmed on the basis of latitude (Fig. 3, Table 3). In the ROK, Karp-related strains were the most predominant and identified in all regions. Kato and Saitama-related strains were mainly identified in the northern and southern regions. Gilliam-related, Boryong, and Shimokoshi strains were identified in the northern, central and southern, and northern and southern regions, respectively. Detailed temporal and geographic information is provided in Additional file 2: Table S2.

**Table 3:** Detection and genotype analysis of *Orientia tsutsugamushi* in chigger mites collected from wild rodents in the Republic of Korea in 2021–2023

Regions 1	Regions 2	Positive	Strains						
			Karp-related	Saitama-related	Boryong	Gilliam-related	Kato-related	Simokoshi	N.D.
North	CW	15	1	3	–	8	3	–	–
	GN	4	–	1	–	–	2	–	1
	HS	2	2	–	–	–	–	–	–
	PJ	13	3	3	–	1	5	–	1
	YJ	3	–	1	–	–	–	1	1
	Subtotal	37	6	8	0	9	10	1	3
Central	CJ	8	4	–	2	–	1	–	1
	BY	2	–	–	2	–	–	–	–
	YS	46	32	1	9	1	1	–	2
	GC	5	–	–	5	–	–	–	–
	YD	2	1	–	1	–	–	–	–
	Subtotal	63	37	1	19	1	2	0	3
South	JE	28	10	6	4	–	2	–	6
	BS	21	1	8	1	–	11	–	–
	JA	3	–	–	–	–	2	1	–
	GJ	–	–	–	–	–	–	–	–
	HC	–	–	–	–	–	–	–	–
	SP	–	–	–	–	–	–	–	–
	Subtotal	52	11	14	5	0	15	1	6
Total		152	54	23	24	10	27	2	12

^*^N.D. not determined

## Discussion

In this study, we investigated the prevalence of *O. tsutsugamushi* and geographical distribution of its strains in chigger mites collected from wild rodents throughout the ROK between 2021 and 2023. A total of 135,204 chigger mites were collected from 1589 wild rodents during the study period, indicating a chigger index of 85.1. Half of the collected chigger mites were divided into 2928 pools to identify *O. tsutsugamushi*, and 152 pools were found to be positive, with an average MIR of 0.22%. Unlike the other years, 2023 had a very low chigger index at 81.7, although we identified 80 positive pools with 0.34% higher MIR. The number of positive pools in 2023 was affected by a sharp increase in the number of positive pools from Yesan (YS), Chungcheongnam-do (Supplementary Material Table S1). Further research is needed to determine the causes of this sudden increase in positivity rates in the area, including environmental and ecological factors. Further, epidemiological analysis should be performed to determine whether it influences the occurrence of scrub typhus.

Here, we identified six types of *O. tsutsugamushi* strains (Fig. 3, Table 3). Previous studies reported that Boryong strains are predominant in chigger mites and patients in the ROK [25, 28]. Although our study may have been influenced by several positive pools from some specific regions, Karp-related strains were the most common, followed by Kato-related, Boryoung, and Saitama-related strains. From an annual perspective, Karp- and Kato-related related strains were the dominant strains in 2021 and 2023 versus 2022, respectively (Supplementary Material Table S2).

Analysis of the geographic distribution of *O. tsutsugamushi* strains confirmed that 54 Karp-related strains were distributed throughout the country, with the highest prevalence in Chungcheong-do, a central region. This pattern differed from previous studies, which reported that Karp-related strains were predominant in northern regions, including Gyeonggi-do and Gangwon-do [33]. Excluding Yeongdeok-gun, most Karp-related strains were distributed in the western region of Korea. Saitama- and Kato-related strains were also found throughout the country, although they were mainly found in the northern and southern regions. The Kato-related strains found in this study shared 99% identity with those of the MZ146364 and MZ146358 sequences reported in an earlier study [1] and 83% identity with those of previously reported Kato-related *O. tsutsugamushi* isolates in GenBank. Such differences were evident in the discovery of new strains distinct from Kato-related strains [41]. Different distribution patterns were also observed for the Boryong and Gilliam-related strains. Herein, we identified 24 Boryong strains mainly distributed in the central and southern regions. Other studies confirmed a similar distribution of the Boryong strains [25, 28, 33, 39]. In contrast, the Gilliam-related strains were mainly distributed in the northern region. We identified ten Gilliam-related strains, most of which were distributed in northern Gyeonggi-do and Gangwon-do, except for Yesan-gun in Chuchungnam-do. A similar distribution pattern for the Gilliam strain was reported by other studies [33, 42]. Herein, two samples from Yeoju-si and Jinan-gun were identified as Simokoshi strains. All five strains, excluding the Simokoshi strain, were confirmed in Yesan-gun. In Geoje-si, Hapcheon-gun, and Seogwipo-si, the number of chigger mites was similar. No positive pools of chigger mites were identified.

The main vectors of scrub typhus in the ROK are *L.* *pallidum* and *L.* *scutellare*, which are distributed mainly throughout the Korean Peninsula and southern regions, respectively [20]. Their distribution is a major determinant of the transmission of Karp and Boryong strains, respectively [25, 28]. However, *L. scutellare* is the main vector of the Karp and Kawasaki strains in China and Japan, respectively [25, 43, 44]. As such, the same species of chigger mites can transmit different strains of *O. tsutsugamushi* depending on the country or region. Thus, the geographical distribution of chigger mites and *O. tsutsugamushi* strains should be investigated. However, some difficulties exist in the individual performance of species identification and infection rate evaluation in large-scale analyses of chigger mites [1]. The geographical distribution of *O. tsutsugamushi* strains in relation to chigger mites was investigated in this study, although the geographical distribution of the chigger mite species itself was not investigated, warranting further research. In addition, over 80–90% of patients with scrub typhus in the ROK are infected with the Boryong strain, whereas the Karp, Gilliam, and Kato strains are reported only in certain areas [45]. Therefore, further research is needed on the differences in strains among patients and chigger mites as well as on how these correlate.

## Conclusions

*Orientia tsutsugamushi* comprises various serotypes and strains depending on the endemic region, with differences in virulence among strains. To prevent and control this disease, it is important to determine the geographic distribution of *O. tsutsugamushi* strains. We summarize the geographical distribution of *O. tsutsugamushi* lineages across Korea from 2021–2023. In chigger mites within the ROK, Karp-related strains of *O. tsutsugamushi* were identified as the most predominant and were mainly distributed in the central region. Gilliam-related strains were found in the northern region, whereas Boryong strains were found in the central and southern regions. Our results provide important data for monitoring and preventing the increase of mite-borne infectious diseases due to globalization and climate change.

## Supplementary Information

Additional file 1. Table S1. *Orientia tsutsugamushi* infection in chigger mites collected from wild rodents in the ROK 2021–2023 (detailed geographic information). Table S2. Detection and genotypes analysis of *Orientia tsutsugamushi* in chigger mites collected from wild rodents in the ROK in 2021–2023 (temporal and geographic information).

## Data Availability

Data supporting the conclusions of this article are included within the article. The newly generated sequences were submitted to the GenBank database under the accession number PQ195687-826. The datasets used and/or analyzed during the current study are available from the corresponding author upon reasonable request.

## Abbreviations

ROK: Republic of Korea
MIR: Minimum infection rate
TSA: Type-specific antigen
NCBI: National Center for Biotechnology Information
