# Supplementary material for: Geographic distribution of Orientia tsutsugamushi strains on chigger mites in the Republic of Korea (2021–2023)
Source: Parasit Vectors. 2025 May 27;18:192. doi: 10.1186/s13071-025-06839-3 (PMC12107758; doi:10.1186/s13071-025-06839-3)
Supplement: Supplementary file 1 — Additional file 1. Table S1. Orientia tsutsugamushi infection in chigger mites collected from wild rodents in the ROK 2021–2023 (detailed geographic information). Table S2. Detection and genotypes analysis of Orientia tsutsugamushi in chigger mites collected from wild rodents in the ROK in 2021–2023 (temporal and geographic information). [file 13071_2025_6839_MOESM1_ESM.docx]

**Table S1**. *O. tsutsugamushi* infection information collected chigger mites from wild rodents in the Republic of Korea 2021–2023 (detailed geographic information)

| **Year** | **Regions** | **No. of rodents infested by chigger mites/No. of rodents (%)** | | **No. of**  **chigger mites** | **Chigger**  **index** | **No. of tested**  **chigger mites**  **(No. of pools)** | **No. of**  ***Ot*^*^ positive**  **chigger**  **mite pools** | **MIR^**^** |
| --- | --- | --- | --- | --- | --- | --- | --- | --- |
| **2021** | CW | 40/42 (95.2) | | 2,591 | 61.7 | 1,306(68) | 7 | 0.54 |
|  | GN | 37/49 (75.5) | | 2,636 | 53.8 | 1,229(61) | 0 | 0 |
|  | YJ | 23/37 (62.1) | | 3,324 | 89.8 | 1,668(71) | 0 | 0 |
|  | GJ | 25/25 (100.0) | | 2,288 | 91.5 | 1,120(52) | 0 | 0 |
|  | HC | 17/25 (68.0) | | 671 | 26.8 | 334(20) | 0 | 0 |
|  | GC | 25/27 (92.6) | | 4,948 | 183.3 | 2,480(94) | 1 | 0.04 |
|  | YD | 19/22 (86.4) | | 2,807 | 127.6 | 1,403(59) | 1 | 0.07 |
|  | HS | 42/50 (84.0) | | 4,653 | 93.1 | 2,311(95) | 2 | 0.09 |
|  | PJ | 23/23 (100.0) | | 3,800 | 165.2 | 1,894(77) | 0 | 0 |
|  | JE | 26/29 (89.7) | | 3,011 | 103.8 | 1,508(58) | 12 | 0.8 |
|  | BS | 17/20 (85.0) | | 2,849 | 142.5 | 1,427(55) | 1 | 0.07 |
|  | JA | 11/28 (39.3) | | 265 | 9.5 | 151(11) | 0 | 0 |
|  | SP | 32/44 (72.7) | | 1,791 | 40.7 | 895(50) | 0 | 0 |
|  | CJ | 10/12 (83.3) | | 1,618 | 134.8 | 810(33) | 0 | 0 |
|  | BY | 14/22 (63.6) | | 710 | 32.3 | 358(20) | 0 | 0 |
|  | YS | 14/16 (87.5) | | 2,253 | 140.8 | 1,130(46) | 9 | 0.8 |
|  | **Sub Total** | **375/471 (79.6)** | | **40,215** | **85.4** | **20,024(870)** | **33** | **0.16** |
| **2022** | CW | 64/67 (95.5) | 6,089 | | 90.9 | 3,061(136) | 6 | 0.2 |
|  | GN | 36/55 (65.5) | 1,699 | | 30.9 | 864(49) | 3 | 0.35 |
|  | YJ | 37/43 (86.0) | 4,121 | | 95.8 | 2,070(87) | 1 | 0.05 |
|  | GJ | 16/25 (64.0) | 2,231 | | 89.2 | 1,114(45) | 0 | 0 |
|  | HC | 19/27 (70.4) | 618 | | 22.9 | 312(25) | 0 | 0 |
|  | GC | 15/18 (83.3) | 1,862 | | 103.4 | 933(40) | 0 | 0 |
|  | YD | 24/26 (92.3) | 3,649 | | 140.3 | 1,829(73) | 0 | 0 |
|  | HS | 26/31 (83.9) | 3,450 | | 111.3 | 1,765(73) | 0 | 0 |
|  | PJ | 41/46 (89.1) | 11,693 | | 254.2 | 5,848(214) | 10 | 0.17 |
|  | JE | 30/30 (100.0) | 3,864 | | 127.0 | 1,949(77) | 4 | 0.2 |
|  | BS | 23/30 (76.7) | 2,489 | | 83.0 | 1,267(55) | 9 | 0.7 |
|  | JA | 23/31 (74.2) | 1,571 | | 50.7 | 788(39) | 0 | 0 |
|  | SP | 23/61 (37.7) | 1,023 | | 16.8 | 514(32) | 0 | 0 |
|  | CJ | 22/24 (91.7) | 2,236 | | 93.2 | 1,120(47) | 6 | 0.5 |
|  | BY | 15/24 (62.5) | 1,075 | | 44.8 | 541(28) | 0 | 0 |
|  | YS | 16/23 (69.6) | 1,832 | | 79.7 | 914(20) | 0 | 0 |
|  | **Sub Total** | **435/561 (77.5)** | **49,502** | | **88.2** | **24,889(1,040)** | **39** | **0.16** |
| **2023** | CW | 36/47 (76.6) | 1,494 | | 31.8 | 754(49) | 2 | 0.27 |
|  | GN | 28/51 (54.9) | 659 | | 12.9 | 340(29) | 1 | 0.29 |
|  | YJ | 45/46 (97.8) | 3,564 | | 77.5 | 1,793(87) | 2 | 0.11 |
|  | GJ | 31/45 (68.9) | 2,597 | | 57.7 | 1,306(62) | 0 | 0 |
|  | HC | 13/19 (68.4) | 2,528 | | 133.1 | 1,264(47) | 0 | 0 |
|  | GC | 22/25 (88.0) | 3,243 | | 129.7 | 1,626(67) | 4 | 0.25 |
|  | YD | 37/38 (97.4) | 5,462 | | 143.7 | 2,738(107) | 1 | 0.04 |
|  | HS | 28/29 (96.6) | 3,826 | | 131.9 | 1,946(79) | 0 | 0 |
|  | PJ | 19/20 (95.0) | 3,833 | | 191.7 | 1,918(72) | 3 | 0.16 |
|  | JE | 18/24 (75.0) | 3,185 | | 132.7 | 1,594(61) | 12 | 0.75 |
|  | BS | 29/36 (80.6) | 6,159 | | 171.1 | 3,106(116) | 11 | 0.35 |
|  | JA | 35/38 (92.1) | 2,243 | | 59.0 | 1,570(61) | 3 | 0.19 |
|  | SP | 33/67 (49.3) | 943 | | 14.1 | 479(52) | 0 | 0 |
|  | CJ | 21/24 (87.5) | 1,288 | | 53.7 | 646(36) | 2 | 0.31 |
|  | BY | 17/24 (70.8) | 1,234 | | 51.4 | 617(28) | 2 | 0.32 |
|  | YS | 22/24 (91.7) | 3,229 | | 134.5 | 1,621(65) | 37 | 2.28 |
|  | **Sub Total** | **434/557 (77.9)** | **45,487** | | **81.7** | **23,318(1,018)** | **80** | **0.34** |
| **Total** | | **1,244/1,589 (78.3)** | **135,204** | | **85.1** | **68,231(2,928)** | **152** | **0.22** |

^*^*Ot* – *Orientia tsutsugamushi*; MIR- Minimum infection rate of chigger mites (No. of *Ot* positive chigger mite pools/No. of tested chigger mites x 100)

**Table S2**. Detection and genotypes analysis of *Orientia tsutsugamushi* in chigger mites collected from wild rodents in the Republic of Korea in 2021–2023 (temporal and geographic information)

| **Year** | **Regions** | **Month** | **Positive** | **Strain** | | | | | | |
| --- | --- | --- | --- | --- | --- | --- | --- | --- | --- | --- |
|  |  |  |  | **Karp-related** | **Saitama-related** | **Boryong** | **Gilliam-related** | **Kato-related** | **Simokoshi** | **N.D** |
| **2021** | CW | 4 | 7 | 1 | 2 | - | 2 | 1 | - | - |
|  |  | 11 |  |  |  |  | 1 | - | - | - |
|  | GC | 11 | 1 | - | - | 1 | - | - | - | - |
|  | YD | 4 | 1 | - | - | 1 | - | - | - | - |
|  | HS | 10 | 2 | 1 | - | - | - | - | - | - |
|  |  | 11 |  | 1 | - | - | - | - | - | - |
|  | JE | 3 | 12 | 1 | - | - | - | - | - | 1 |
|  |  | 4 |  | 1 | 1 | - | - | - | - | - |
|  |  | 10 |  | 1 | - | 1 | - | - | - | - |
|  |  | 11 |  | 5 | - | - | - | - | - | 1 |
|  | BS | 4 | 1 | - | - | - | - | 1 | - | - |
|  | YS | 3 | 9 | - | 1 | - | - | - | - | - |
|  |  | 4 |  | 3 | - | - | - | - | - | - |
|  |  | 10 |  | 5 | - | - | - | - | - | - |
|  | **Sub Total** | | **33** | **19** | **4** | **3** | **3** | **2** | **0** | **2** |
| **2022** | CW | 3 | 6 | - | - | - | - | 1 | - | - |
|  |  | 4 |  | - | - | - | 3 | 1 | - | - |
|  |  | 11 |  | - | 1 | - | - | - | - | - |
|  | GN | 10 | 3 | - | 1 | - | - | 2 | - | - |
|  | YJ | 3 | 1 | - | - | - | - | - | - | 1 |
|  | PJ | 10 | 10 | 1 | 2 | - | 1 | 3 | - | 1 |
|  |  | 11 |  | 2 | - | - | - | - | - | - |
|  | JE | 3 | 4 | 2 | - | - | - | 1 | - | - |
|  |  | 11 |  | - | - | - | - | 1 | - | - |
|  | BS | 3 | 9 | - | - | - | - | 1 | - | - |
|  |  | 10 |  | - | 2 | - | - | - | - | - |
|  |  | 11 |  | - | 4 | - | - | 2 | - | - |
|  | CJ | 4 | 6 | 1 | - | - | - | - | - | - |
|  |  | 10 |  | 3 | - | - | - | 1 | - | 1 |
|  | **Sub Total** |  | **39** | **9** | **10** | **0** | **4** | **13** | **0** | **3** |
| **2023** | CW | 10 | 2 | - | - | - | 1 | - | - | - |
|  |  | 11 |  | - | - | - | 1 | - | - | - |
|  | GN | 4 | 1 | - | - | - | - | - | - | 1 |
|  | YJ | 3 | 2 | - | - | - | - | - | 1 | - |
|  |  | 10 |  |  | 1 | - | - | - | - | - |
|  | GC | 10 | 4 | - | - | 3 | - | - | - | - |
|  |  | 11 |  | - | - | 1 | - | - | - | - |
|  | YD | 4 | 1 | 1 | - | - | - | - | - | - |
|  | PJ | 10 | 3 | - | 1 | - | - | - | - | - |
|  |  | 11 |  | - | - | - | - | 2 | - | - |
|  | JE | 3 | 12 | - | - | 2 | - | - | - | - |
|  |  | 4 |  | - | - | 1 | - | - | - | - |
|  |  | 11 |  | - | 5 | - | - | - | - | 4 |
|  | BS | 3 | 11 | 1 | 1 | - | - | 1 | - | - |
|  |  | 4 |  | - | - | - | - | 3 | - | - |
|  |  | 10 |  | - | - | 1 | - | 1 | - | - |
|  |  | 11 |  | - | 1 | - | - | 2 | - | - |
|  | JA | 10 | 3 | - | - | - | - | 2 | 1 | - |
|  | CJ | 11 | 2 | - | - | 2 | - | - | - | - |
|  | BY | 3 | 2 | - | - | 1 | - | - | - | - |
|  |  | 4 |  | - | - | 1 | - | - | - | - |
|  | YS | 3 | 37 | 7 | - | - | - | - | - | - |
|  |  | 4 |  | 2 | - | 9 | 1 | - | - | 1 |
|  |  | 10 |  | 8 | - | - | - | 1 | - | - |
|  |  | 11 |  | 7 | - | - | - | - | - | 1 |
|  | **Sub Total** | | **80** | **26** | **9** | **21** | **3** | **12** | **2** | **7** |
| **Total** | |  | **152** | **54** | **23** | **24** | **10** | **27** | **2** | **12** |

^*^N.D. – not determined
